# Cable‐Car Electrocatalysis to Drive Fully Decoupled Water Splitting

**DOI:** 10.1002/advs.202301872

**Published:** 2023-07-03

**Authors:** Yuanzheng Long, Cheng Yang, Yulong Wu, Bohan Deng, Ziwei Li, Naveed Hussain, Kuangyu Wang, Ruyue Wang, Xian He, Peng Du, Zeliang Guo, Jialiang Lang, Kai Huang, Hui Wu

**Affiliations:** ^1^ State Key Lab of New Ceramics and Fine Processing School of Materials Science and Engineering Tsinghua University Beijing 100084 China; ^2^ Center of Advanced Mechanics and Materials Applied Mechanics Laboratory Department of Engineering Mechanics Tsinghua University Beijing 100084 China; ^3^ State Key Laboratory of Information Photonics and Optical Communications & School of Science Beijing University of Posts and Telecommunications Beijing 100876 China

**Keywords:** cable car electrodes, hydrogen production, nickel hydroxide, water electrolysis, water splitting

## Abstract

The increasing demand for clean energy conversion and storage has increased interest in hydrogen production via electrolytic water splitting. However, the simultaneous production of hydrogen and oxygen in this process poses a challenge in extracting pure hydrogen without using ionic conducting membranes. Researchers have developed various innovative designs to overcome this issue, but continuous water splitting in separated tanks remains a desirable approach. This study presents a novel, continuous roll‐to‐roll process that enables fully decoupled hydrogen evaluation reaction (HER) and oxygen evolution reaction (OER) in two separate electrolyte tanks. The system utilizes specially designed “cable‐car” electrodes (CCE) that cycle between the HER and OER tanks, resulting in continuous hydrogen production with a purity of over 99.9% and Coulombic efficiency of 98% for prolonged periods. This membrane‐free water splitting system offers promising prospects for scaled‐up industrial‐scale green hydrogen production, as it reduces the cost and complexity of the system, and allows for the use of renewable energy sources to power the electrolysis process, thus reducing the carbon footprint of hydrogen production.

## Graphical abstract

In this work, a continuous “cable car” process is developed to separate the hydrogen and oxygen production processes. This design successfully produces hydrogen with 99.9% purity without using expensive membranes for >10 h. Apart from being membrane‐free, this unique system offers promising potential for scaled‐up industrial‐scale green hydrogen production.

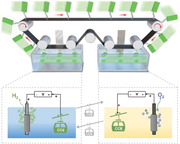

## 1 Introduction

The need to reduce carbon emissions has become a pressing concern as global industries and economic activities continue to expand.^[^ ^1^ ^]^ To address this issue, hydrogen is being viewed as a next‐generation energy source due to its zero‐carbon emissions, non‐toxic byproducts, and high energy density per unit mass.^[^ ^2^ ^]^ Various methods of producing hydrogen, such as thermochemical reform, electrolytic, photolysis, and photo‐electrolytic water splitting, have been extensively studied.^[^ ^3^ ^]^ However, traditional hydrogen production methods, such as grey hydrogen produced by the chemical reforming of fossil fuels, cause significant carbon emissions.^[^ ^4^ ^]^ In contrast, green hydrogen produced by electrolysis using renewable electricity is gaining attention as a sustainable and efficient technique for hydrogen production with reduced carbon emissions.^[^ ^5^ ^]^ Renewable energy sources, such as solar, wind, and tidal energies, are especially suitable for producing green hydrogen as they provide intermittent electricity.^[^ ^6^ ^]^

In a typical electrolytic water‐splitting process, hydrogen and oxygen are produced by electrochemical reactions at separate electrodes. However, this process also results in the production of a mixed gas of H_2_/O_2_, which poses a significant challenge to the efficient utilization of hydrogen. To overcome this challenge, conventional water‐splitting equipment employs ion‐exchange membranes or ion‐permeable separators, such as proton exchange membranes or anion exchange membranes, to prevent undesirable ion or gas crossover.^[^ ^3^, ^7^ ^]^ In these conventional systems, the two electrodes are immersed in the same device with an aqueous electrolyte, and an ion‐exchange membrane or porous diaphragm separates the two different reaction zones. However, using these components can reduce hydrogen production efficiency and the overall energy efficiency.^[^ ^8^ ^]^ Additionally, the high cost of these membranes and their vulnerability in high‐pressure scenarios and long‐term operation raises economic and safety concerns for practical applications.^[^ ^6^, ^9^ ^]^

Researchers in the field of energy are currently exploring new and innovative designs for decoupled water‐splitting techniques, which aim to separate the production of hydrogen and oxygen without the use of ionic conducting membranes. These designs include using liquid mediators in redox‐flow batteries to produce hydrogen and oxygen non‐spontaneously, as well as using auxiliary electrodes that absorb and release ions to delay the spontaneous emission of hydrogen and oxygen. In all cases, hydrogen and oxygen have been produced either within the same tank or by using an intermittent pulse to replace the auxiliary electrodes.^[^ ^10^ ^]^ These designs aim to produce purer hydrogen with fewer gaseous byproducts. One example of such design is the use of a redox mediator to decouple water by Rausch et al.^[^ ^8a^ ^]^ Another example is the design proposed by Chen, Landman, and Dotan, which incorporates chemicals such as nickel hydroxide into the reaction and develops several devices to enhance hydrogen production.^[^ ^10^, ^11^ ^]^

These devices are well‐constructed and integrate the distinctive electrochemical properties of redox mediators with the concept of decoupled water splitting, which separates the hydrogen evolution reaction (HER) and oxygen evolution reaction (OER) processes.^[^ ^6^, ^8^, ^11^ ^]^ They achieved this by combining thermal reduction, photovoltaic cells, zinc anode, or other designs to produce hydrogen from a single tank for a specified period, followed by changing electrodes or replacing the consumed materials. The combination of hydrogen production electrochemical reactions with other electrochemical, chemical, thermally activated chemical, and photoelectrochemical reactions provides credible alternatives to traditional water electrolysis.^[^ ^12^ ^]^ These studies outlined feasible strategies for maximizing the use of wasted water, seawater, and renewable energy.^[^ ^13^ ^]^

These innovative designs provide benefits to innovative water‐splitting devices, including spatial and temporal separation, safety, modularity, and sustainability.^[^ ^10^, ^14^ ^]^ Further improvements are expected to ensure the durability and stability of electrodes and devices for future sustainable application.^[^ ^10^, ^15^ ^]^ Also, membranes and key components related to them occupy more than 15% of the electrolytic stacks in the alkaline electrolyzer or more than 24% of the electrolytic stacks in the proton exchange membrane electrolyzer.^[^ ^16^ ^]^ Avoiding the usage of membranes can genuinely reduce the cost of constructing an electrolyzer. Despite the potential for continuous hydrogen production, existing designs have limitations regarding mechanical maintenance and the need for intermittent suspensions to exchange hydrogen‐generation and oxygen‐generation tanks. Safe, stable, and continuous designs utilizing auxiliary mediators to produce pure hydrogen without expensive membranes are still eagerly expected.^[^ ^17^ ^]^

In this study, we present a novel device capable of continuous hydrogen production using a roll‐to‐roll process and a unique “cable‐car” electrode (CCE) design. By utilizing the special chemical properties of nickel (oxy)hydroxide, we were able to completely separate the hydrogen evolution reaction (HER) and oxygen evolution reaction (OER) in our system. This allowed for pure hydrogen production with prolonged stability and high Coulombic efficiency. Additionally, our design eliminates the need for costly membranes, making it a cost‐effective solution for hydrogen generation. The modular and simple design of the device also allows for ease of maintenance and electrode replacement, making it a viable option for future large‐scale hydrogen production. Furthermore, the system's robustness enables it to operate with low‐quality or intermittent power sources, making it suitable for renewable energy sources.

## 2 Design of CCEs and Roll‐To‐Roll Process

This study presents a novel decoupled water‐splitting electrolysis system that utilizes auxiliary electrodes and a membrane‐free design. Our approach enables the continuous production of ultra‐pure hydrogen in one tank, while oxygen production and the recovery of cable‐car electrode materials are carried out in the other tank, using roll‐to‐roll transfer of nickel hydroxide (Ni(OH)_2_) cable‐car electrodes (CCE). The Ni^(II)^↔Ni^(III)^ reaction pairing on the CCEs allows for an intermediary state that avoids the direct production of oxygen.^[^ ^8^, ^10^, ^11^ ^]^ The CCEs are designed to operate in roll‐to‐roll cycles, enabling the continuous production of hydrogen without the use of expensive membranes.^[^ ^10^, ^11^, ^18^ ^]^ This continuous reaction process with CCEs has the potential to be applied to other cascade reactions, such as ethanol and starch production or wastewater treatment.^[^ ^19^ ^]^

**Figure 1:**
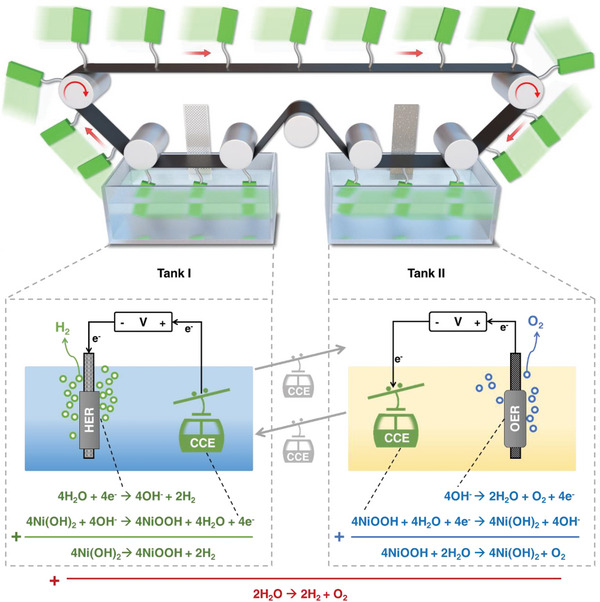
Design of CCE water splitting system. The schematic illustration of the CCE system and the reaction design. CCEs are rolled from one tank to the next, producing hydrogen in Tank I and oxygen in Tank II. This system offers continuous hydrogen production. The mechanical transfer by the stainless‐steel conveyor separates the hydrogen production and oxygen production processes. The detailed reaction process is shown with chemical equations.

The hydrogen production device (illustrated in **Figure** **1**) consists of several modules: a stainless‐steel conveyor module that conveys the CCEs, current, and momentum; a hydrogen production and collection (HER) module; and an electrode recovery (OER) module. Detailed information about the construction of our device can be found in the supporting information. The stainless‐steel conveyor belt with numerous electrodes emerged as the main module for driving the reaction, as shown in Figure 1.

(1) $$4Ni^{\left(\mathrm{II}\right)}{\left(\mathrm{OH}\right)}_{2}\to 4Ni^{\left(\mathrm{III}\right)}\mathrm{OOH}+2H_{2}$$

(2) $$4Ni^{\left(\mathrm{III}\right)}\mathrm{OOH}+2H_{2}O\to 4Ni^{\left(\mathrm{II}\right)}{\left(\mathrm{OH}\right)}_{2}+O_{2}$$

(3) $$2H_{2}O↔2H_{2}+O_{2}$$

**Figure 2:**
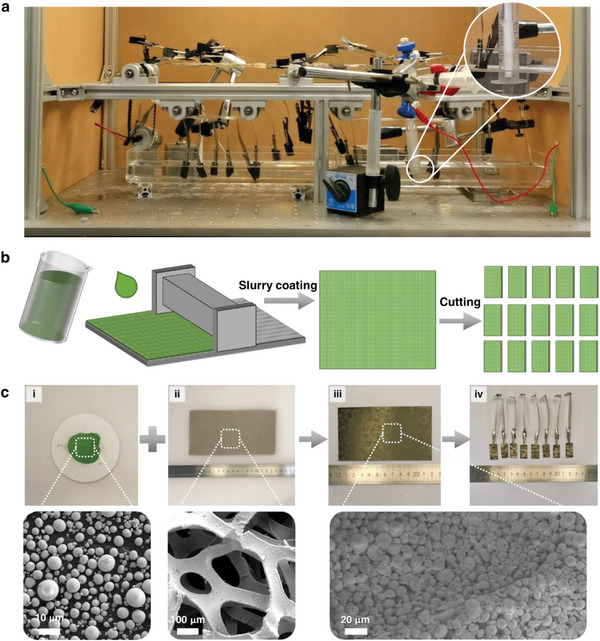
Construction of the CCEs and the electrolysis system. a) A digital photograph of the CCE system. Inset: digital image of the collection module during hydrogen production. Hydrogen bubbles and a decreased liquid level in the syringe are observed. Videos S1 and S2 (Supporting Information) provide videos describing the operation of the system. b) A schematic diagram of the slurry coating method to prepare CCEs. This method enables large‐scale electrode production. c) The digital and SEM images of the Ni(OH)_2_ powder (i), foam nickel (ii), and CCEs (iii, iv).

**Figure 3:**
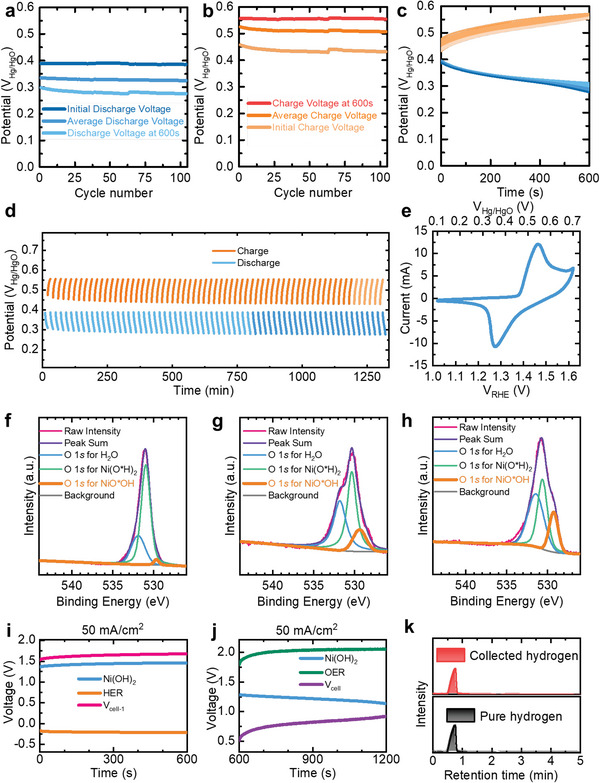
Chemical and electrochemical performance of the CCEs and the running system. a–d) The electrochemical performance of a CCE. a) Stable discharging performance for over 100 cycles. The average discharging potentials of each cycle remain stable, and the potential difference during 600 s remains ≈0.1 V. b) Stable charging performance for over 100 cycles. The average charging potentials of each cycle remain stable, and the potential difference during 600 s remains ≈0.1 V. c) Overlapping charging and discharging curves for over 100 cycles, indicating the feasibility of applying these CCEs into the operating system. The orange and blue lines refer to the charging and discharging processes, respectively. d) Electrochemical cycling performance of a CCE for over 100 cycles. The orange and blue lines refer to the charging and discharging processes, respectively. e) The current‐potential curve of Ni(OH)_2_. The peak at ≈0.55 V versus Hg/HgO is for the reaction from Ni^(II)^ to Ni^(III)^. f–h) XPS analysis of the CCE in different stages: f) initial stage, g) ≈50% charged, h) fully charged. The rising orange peak indicates the appearance of NiO*OH, which provides evidence of our reaction theory. i) Voltage relationships during the charging process, where HER coincides. j) Voltage relationships during the discharging process, where OER coincides. k) Gas chromatography (GC) results for the collected and pure hydrogen samples. The GC result shows an over 99.9% purity of obtained hydrogen.

In an alkaline medium of 1 m KOH, we applied a voltage greater than 1.4 V to drive the reaction of hydrogen production (Equation 1). Conversely, a voltage less than 0.9 V was applied to drive the reaction of oxygen production (Equation 2, Figure 3e, and Figure S1, Supporting Information).^[^ ^20^ ^]^ Our research builds upon previous studies, which have demonstrated the reversibility of this reaction as an appropriate mediator for decoupled water splitting. ^[^ ^10^, ^11^, ^21^ ^]^ Based on this understanding, we developed electrodes composed of this material to separate water splitting into two distinct reactors. Through slurry coating, we were able to maximize the production of nickel hydroxide on nickel foam (**Figure** **2**c).

In our design, we have separated the traditional water‐splitting reaction (Equation 3) into two separate tanks. The first tank is dedicated to hydrogen production, utilizing a nickel hydroxide‐covered electrode that undergoes a reaction from Ni^(II)^ to Ni^(III)^, allowing for simultaneous hydrogen production. The electrodes are then transferred to the second tank via a conveyor belt and reduced from Ni^(III)^ to Ni^(II)^ for oxygen production.^[^ ^11^ ^]^ Our design utilizes the nickel hydroxide electrode as an auxiliary electrode to transfer hydrogen or oxygen atoms between the tanks, eliminating the need for traditional membrane or diaphragm separations. However, it is important to maintain a specific voltage range to prevent the oxygen evolution reaction (OER) from taking place, as the OER can occur if the electrode is charged over 100 C cm^−2^. Overall, this technique allows for the separation of hydrogen and oxygen production into two separate tanks, enabling the production of pure hydrogen with no H_2_/O_2_ crossover by utilizing the transformation between Ni^(II)^(OH)_2_ and Ni^(III)^OOH.

To evaluate the stability of our electrodes, we conducted additional electrochemical testing. We submerged the electrodes in each tank for 10 min to simulate the actual operating conditions and found that the HER and OER processes occurred independently in the two tanks for over 100 cycles (10 min charged and 10 min discharged for each cycle). We recorded the electrochemical performance of HER and OER cycling using a standard electrode designed for this purpose (**Figure** **3**a–d). The results showed a stable oxidized potential (≈0.50 V vs Hg/HgO) for the hydrogen production process (Equation 1) and a reduced potential (≈0.35 V vs Hg/HgO) for the oxygen production process (Equation 2). The potential fluctuated by ≈0.10 V every 10 min during each charging and discharging process, while the overpotential remained at an average level.

On the other hand, our results showed that the charging and discharging potentials remained stable during their operational periods. Furthermore, the sequential charging and discharging curves presented in Figure 3c support our conclusion. It should be noted that during actual operation, a single nickel hydroxide electrode lasted no longer than 10 min in one reaction tank. Therefore, the electrochemical status changes during the production process can be effectively evaluated using the intermittent stimulation method. The charging and discharging curves showed consistency for over 100 cycles, indicating the excellent electrochemical stability of the electrodes. To further demonstrate the stability of the electrodes, we also investigated their complete charge and discharge performance (Figure S2, Supporting Information). The results showed that the coulombic efficiency of these materials remained at ≈100% for more than 50 cycles, which highlights the potential for their use in various applications.

Our results provide compelling evidence for the practicality of our design. First, the electrodes maintained a constant potential during the charging and discharging process. In combination with the other results, this verifies that the electrodes would have relatively stable charging and discharging potentials in our prototype, which has been designed to operate in less than 10 min for each process.^[^ ^10a,b^ ^]^ This time can be extended by increasing the surface area of the electrodes or by applying a smaller current. Second, it has also been demonstrated that the electrodes were stable. These results indicate that with the same charging and discharging time, the electrodes’ electrochemical status remained consistent, as illustrated in Figure S3 (Supporting Information). A similar phenomenon, which has been previously studied, ensures that every electrode in the entire facility can be cycled effectively. The state of charge should be considered a reliable approach to evaluate the electrochemical status of the electrodes because the phase variance or valence difference during this process is very small. Third, despite their minimal disposition, electron transitions were observed via X‐ray photoelectron spectroscopy (XPS) before and after being charged to certain states of charge (Figure 3f–h; Figure S4, Supporting Information). The most significant increase of the orange peak in Figure 3f–h indicates the transformation from Ni(OH)_2_ to NiOOH. The difference in nickel spectra was not particularly prominent, but a slight shift in the Ni peak could still be observed. These spectroscopy results matched the observation of the state of charge, providing persuasive evidence for the fundamental theory of our design.

## 3 Fully Decoupled Water Splitting with CCEs

The design was inspired by roller coasters such as carousels and cable cars. Instead of using expensive membranes to separate produced gas or produce hydrogen intermittently, we employed a conveyor belt to transport the CCEs between different modules to accomplish decoupled water splitting.^[^ ^10^, ^22^ ^]^ Nickel hydroxide‐coated CCEs were connected to a stainless‐steel conveyor belt to provide mechanical momentum and ensure consistent potential. The conveyor was able to split the two reaction steps for each electrode into two tanks by utilizing the unique chemical properties of nickel hydroxide and the cycling movements.^[^ ^10a,f^ ^]^

A complete cycle in our prototype design involved the following steps: 1) immersion of one electrode in the first reaction tank with an HER counter electrode for hydrogen production, 2) uplift from the first tank, 3) immersion in the second reaction tank with an OER counter electrode for electrode recovery, 4) lifting from the second tank, and 5) position resetting by the conveyor. Continuous hydrogen production and the migration from Ni^(II)^ to Ni^(III)^ proceeded on each CCE consecutively and regularly, while oxygen production and the reduction from Ni^(III)^ to Ni^(II)^ occurred in the second tank. The whole process is illustrated in Videos S1 and S2 (Supporting Information). The electrochemical workstation provided a 50 mA constant current in the first tank, corresponding to an ≈1.6 V_cell_ voltage for hydrogen production (Figure 3i). In this design, a constant voltage of <1.6 V is sufficient to drive the HER process in the first tank. By separating hydrogen production from oxygen production, the likelihood of forming a mixture of gaseous products or other undesired byproducts is minimal, even if the operating voltage experiences occasional fluctuations. A 50 mA constant current in the second tank was used as the proof‐of‐concept experiment, corresponding to a voltage of ≈0.75 V_cell_ for oxygen production (Figure 3j). A constant voltage of 0.80 V was applied to our prototype equipment as it allowed for the reaction to proceed with an applied voltage of less than 1.0 V. In the future, electricity from low‐quality intermittent supply or unstable voltage from renewable sources could be used for large‐scale production. Other operating current densities were also tested to determine the potential relationships between these components (Figure S5, Supporting Information).

**Figure 4:**
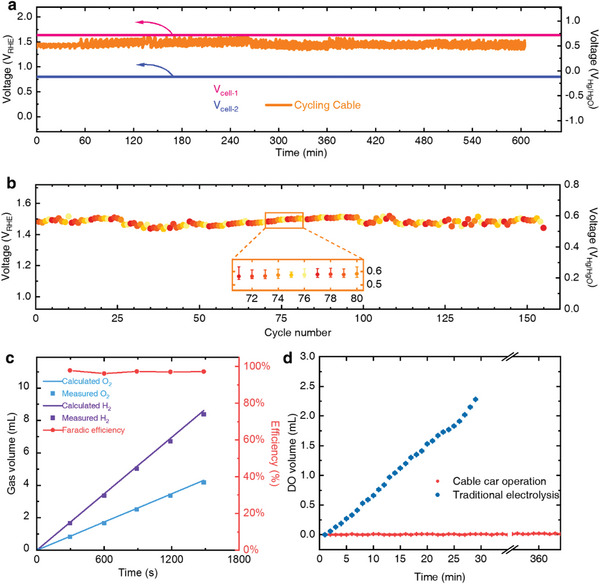
Long‐term hydrogen production. a) Long cycling performance of the running CCE system for over 10 hours. Magenta and blue lines mark the average V_cell‐1_ and V_cell‐2_, respectively. b) Average operating voltage of the device for over 10 h (inset: the voltage ranges from the 71st to the 80th cycles). The voltage changes within ≈100 mV. c) The volume and efficiency of gas production. The measured volume of produced hydrogen and oxygen to meet the calculated theoretical volume. d) Dissolved oxygen (DO) measurement in the HER tank (red). When applying traditional electrolysis (hydrogen and oxygen emit from the same tank), the dissolved oxygen keeps rising in the tank. The dissolved oxygen concentration does not increase during a 6 h measurement when our CCE approach is used.

Continuous hydrogen production was observed and examined to prove the feasibility of our device. A specially designed gas collection module (Figure S6, Supporting Information) has been used to collect hydrogen in our prototype, as illustrated in Figure 2a and Video S2 (Supporting Information). In larger‐scale applications, collection modules with gas pumps or tubes could also be used. Gas chromatography (GC) revealed 99.93% purity of the collected hydrogen (Figure 3k). The absence of oxygen in the collected gas ensured the safety and practicality of using the produced hydrogen. Furthermore, dissolved oxygen (DO) tests revealed that the DO in the first tank did not significantly increase despite the CCEs being continually immersed in and lifted from it (**Figure** **4**d).

We operated our prototype device for over 10 hours at a constant current of 50 mA to prove its operational stability. Figure 4a shows that the electrode potential remained around 0.6 V versus Hg/HgO. The pink and blue lines depicted the voltage of the two cells, as determined by the voltage relations, which can be seen in Figure 3i. To maintain mechanical and electrochemical stability, the conveyor revolved for approximately 240 seconds per cycle. The rotating speed of the conveyor belt can be adjusted to meet practical requirements because this design is highly intuitive and modularized. The slight variation in potential cycle ranges (Figure 4b) provides cycling stability and prolonged cycle feasibility. The operating voltage can fluctuate due to mechanical movements or the altering distance between CCEs and counter electrodes. The faradic efficiency of hydrogen production can be nearly 100% by measuring the volume of hydrogen produced (Figure 4c). According to the data collected from this prototype, our design was found to be stable and efficient. The applicability of our idea of combining mechanical movement with the unique electrochemical properties of mediator materials has been verified.

## 4 Continuous and Massive Hydrogen Production

Our prototype design offers a potential solution for large‐scale hydrogen production, demonstrating continuous ultrapure hydrogen generation with high faradic efficiency (Figure 4c) and promising stability. By incorporating mechanical movement and decoupling the water‐splitting process, our method has the potential to significantly reduce the cost of hydrogen production when scaled up. The use of nickel hydroxide in subsequent studies has shown its ability to withstand high currents (Figure S5, Supporting Information) without releasing oxygen simultaneously, suggesting that scaling up the design by increasing the surface area of each CCE can enhance the overall operating current. In fact, by implementing larger “cable cars” with increased surface area, we can substantially increase the yield rate of hydrogen production. Manipulating the composition of the electrodes, specifically through the incorporation of Ni(OH)_2_ doping, holds the promise of reducing the required voltage and improving the overall efficiency of the system. Additionally, adjusting the temperature, particularly in the OER tank, facilitates the increased involvement of active materials, thereby further enhancing the performance of the system.^[^ ^10b^ ^]^

One notable advantage of our design is the absence of H_2_/O_2_ crossover in either tank, making hydrogen collection more convenient compared to traditional designs.^[^ ^10b^ ^]^ With no expensive membrane required in the hydrogen production tank, and mechanical pumps utilized for continuous gas transport, our system offers a cost‐effective and practical solution. For applications requiring ultrapure hydrogen, a hermetically sealed hood or box enclosing the entire equipment can be purged with nitrogen or other inert gas before operation. Collecting the oxygen produced in the other tank helps prevent leakage or cross‐contamination from the environment.

Our modular design, inspired by roll‐to‐roll systems commonly employed in industrial settings, ensures scalability and straightforward implementation. The architecture of our system not only enables convenient maintenance but also ensures uninterrupted operation, even in demanding real‐industry conditions. Furthermore, the decoupled water splitting approach employed in our system underscores its compatibility with sustainable energy sources, demonstrating the system's capability to withstand irregular power supply and adjust rotating speed without incurring undesirable side reactions.

These advantages pave the way for a more environmentally‐friendly approach to large‐scale hydrogen production, which can be further enhanced by incorporating wastewater, seawater, and solar energy.^[^ ^1^, ^10^, ^13^, ^23^ ^]^ Additionally, the concept of roll‐to‐roll CCEs holds potential for applications beyond hydrogen production, such as battery reactions, bio‐electrochemical systems, pollutant reduction, ethanol production, and other cascade reactions.^[^ ^19^ ^]^

## 5 Conclusion

In summary, this paper presents a fully decoupled electrocatalytic water‐splitting system with a novel electrode design inspired by cable cars. We achieved continuous hydrogen production with a purity of over 99.9% for more than 10 h. Although we used centimeter‐scale CCEs as the prototype to demonstrate the concept in our report, the operating current and hydrogen production rate could be significantly increased by expanding the dimensions of CCEs to a larger scale. Since this design is intuitive and extensible, the hydrogen production rate and efficiency could be enhanced if scaled up to an industrial level. Additionally, due to the separation of modules, a low‐quality and fluctuating power supply would not disrupt the operation of our roll‐to‐roll process, as demonstrated by our results. This water electrolysis system can be deployed in power plants that provide residual energy or intermittent electricity. We believe that this design presents a sustainable path for the effective utilization of renewable energy in the future. Additionally, the design of roll‐to‐roll running CCEs may also assist in other frontier cascade reactions or the chemical production of other industrial assembly lines.

## 6 Experimental Section

### Preparation of the Electrodes

The self‐made Ni(OH)_2_ electrodes were prepared with the slurry coating method. PVDF (poly(vinyldene fluoride), Aldrich, 99%) was dispersed in NMP (N‐Methylpyrrolidone, TCI, 99.9%) to get a 2% PVDF‐NMP solution. Ni(OH)_2_ (nickel hydroxide, Aladdin, 99.9%), SBR (Polymerized Styrene Butadiene Rubber, NV7218, HLLD Co., Ltd, 99%), and carbon black (C45, MTI Group) (at a 10:1:2 mass ratio) were added into the 2% PVDF‐NMP solution. The resulting suspension was then stirred for 4 h at ambient temperature to a slurry state. The resulting slurry was then applied to a nickel foam plate (30×20×0.1 cm). A blade coater was used to coat the slurry evenly onto the nickel foam. The coated nickel foam was dried at 80 °C for twelve hours and then cut into 1×2×0.1 cm electrodes for the HER experiment. This method allowed the massive production of Ni(OH)_2_ electrodes. The purchased electrodes were bought from Helve Lida Co., LTD (China). [hlldpower.com]

The Ir/C electrode for the OER process was prepared as follows. Pure water, ethanol, and Nafion (at an 8:1:1 mass ratio) were mixed into a 2 mL solution. Five milligrams 20% Ir/C (Premetek) was added to the resulting solution. The suspension was mixed with stirring and ultrasonication for 20 min and then dropped onto conductive carbon paper (1 cm × 2 cm). After drying at ambient temperature, the carbon paper can be cut into 1×2 cm electrodes for the OER experiment.

### Fabrication and Operation of the Roll‐To‐Roll Device

The roll‐to‐roll device was made of the main module with a conveyor belt and several “cable car” reactor units, reaction tank modules, gas collection modules, a driving structure, and a supporting scaffold.

Every reactor unit was connected to the stainless steel conveyor belt by welding the conveyor belt and a fusiform stainless tape. The other end of the tape was welded with an alligator clip to clip the 1×2 cm Ni(OH)_2_ electrode. Two quartz tanks were beneath the conveyor belt containing 1 m KOH aqueous solution (Codow, 99.99%). The conveyor belt was shaped to a specific form by the stainless steel wheels and gears, allowing the attached reaction units to dip into and rise out of the quartz tanks. The driving structure was composed of an electric motor, roller bearings, stainless steel wheels, and gears (purchased from local hardware stores). All components of the driving structure were fixed on a custom‐made stainless steel scaffold.

During the operation period, momentum was conducted from the electric motor through the gears and wheels to the stainless steel conveyor belt with reactor units. Each drooping reactor unit was dipped into the first quartz tank until totally immersed in the solution and experienced the first reaction. After the first reaction, it was risen from the first quartz tank and then dipped into the second quartz tank for the reduction reaction. All reactor units went in and out between two quartz tanks subsequently and periodically.

The gas collection module includes a Pt electrode, two three‐way valves, a 10‐mL syringe, a 20‐mL syringe, and a gas collecting bag. The custom‐made Pt electrode with a long wire was enshrouded under a 10‐mL syringe. One end of the lower three‐way valve was connected to the 10‐mL syringe for conducting the current and the emitted gas. Another end of the lower valve allowed the exit of the long wire connected to the custom‐made Pt electrode, which was sealed with Tack‐It to ensure seamlessness. The rest end of the lower valve was connected to another three‐way valve (the upper valve), which connected a 20‐mL syringe and a gas collecting bag through a polyurethane tube (or through a gas booster pump if necessary). The connections were all seamless with no air leaks, which were intentionally tested before the operation.

Before the operation period, nitrogen was purged into quartz tanks, two syringes, and the gas collecting bag to eliminate the dissolved and residual oxygen. The upper valve was adjusted only to allow passage from the 20‐mL syringe and the lower valve. The residual gas (mostly nitrogen) was extracted from the 20‐mL syringe with the rising liquid level in the 10‐mL syringe. The upper valve was then adjusted to block the passage from the lower valve. The extracted gas in the 20‐mL syringe was released into the environment.

During the operation period, the emitted gaseous product from the Pt electrode was collected in the 10‐mL syringe. Two methods could be applied to collect the emitted gaseous product. One possible way was to intermittently extract the gaseous product through the upper valve and the 20‐mL syringe (the upper valve should only allow the passage between the lower valve and the 20‐mL syringe at this time). Then the gaseous product was injected into the gas‐collecting bag with its own valve (the upper valve should only allow the passage between the 20‐mL syringe and the gas‐collecting bag at this time). This method is recommended for sample testing or temporary storage. Another way was to directly extract the gas through a gas booster pump to the gas collecting bag or characterization machines (the upper valve should only allow the passage between the lower syringe and the pump at this time).

### Electrochemical Measurement

The electrochemistry tests were conducted by a CHI760 electrochemistry workstation (CHI760E, Shanghai Chenhua Instruments). Current‐voltage curves of Ni(OH)_2_ in Figure 2e were tested at 50 mV s^−1^ from 0.1 V to 0.7 V versus V_Hg/HgO_ (i.e., 1.02 V to 1.62 V versus V_SHE_ at pH 14). Long cycling performance curves in Figure 2a‐d were tested by an alternate chronopotential (CP) mode with a consecutive 600 s charging process at 50 mA and a following 600 s discharging process at 50 mA. The voltage relations between electrodes in Figure 3i,j were discovered by CP tests with charging and discharging current at 50 mA for 600 s. The long‐time running results in Figure 4 were recorded in a CP test with a constant 50 mA charging current. All proof‐of‐concept experiments were conducted with a 1×2 cm Ni(OH)_2_ electrode (only 1 cm of the electrode was immersed in the solution). The reference electrode was a Hg/HgO standard electrode (R501, Shanghai Yueci). The counter electrode in the HER process was a Pt mesh (1×1 cm) electrode (99.99%, Shanghai Yueci). During the operation period, a constant voltage from a DC power (GPS‐2303C GWINSTEK) was applied to the operating module between the Ir/C electrode and the reaction units.

### Characterization

The microstructure of all the samples was investigated by scanning electron microscopy with a MERLIN Compact Zeiss scanning electron microscope (SEM). TEM observations were conducted on JEOL‐ARM‐200F TEM operated at 200 kV. The X‐ray diffraction (XRD) patterns of the as‐fabrication materials were evaluated using a D/max‐2500 diffractometer (Rigaku, Japan) equipped with a CuK*α* radiation source. The X‐ray photoelectron spectroscopy (XPS) pattern of the materials was evaluated using a Thermo Fisher spectrometer (Escalab 250Xi) equipped with an Al K*α* radiation source (1487.6 eV) and hemispherical analyzer with a pass energy of 30.0 eV and energy step size of 0.05 eV. The volume of the collected gaseous product was calculated considering the marks on the 10‐mL syringe and the volume of the Pt electrode. A GC‐2014 Shimadzu gas chromatograph conducted the gas chromatography (GC) tests. The gas purity was determined by comparing the integral areas of the gas chromatograph. The dissolved oxygen (DO) was measured using a DO probe (SX725, SANXIN).

## Conflict of Interest

The authors declare no conflict of interest.

## Supporting information

Supporting Information

Supplemental Video 1

Supplemental Video 2

## Data Availability

The data that support the findings of this study are available from the corresponding author upon reasonable request.
