# Supplementary material for: Cable‐Car Electrocatalysis to Drive Fully Decoupled Water Splitting
Source: Adv Sci (Weinh). 2023 Jul 3;10(26):2301872. doi: 10.1002/advs.202301872 (PMC10502859; doi:10.1002/advs.202301872)
Supplement: Supplementary file 1 — Supporting Information [file ADVS-10-2301872-s003.pdf]

ADVANCED SCIENCE
Open Access

Supporting Information

for *Adv. Sci.*, DOI 10.1002/advs.202301872

Cable-Car Electrocatalysis to Drive Fully Decoupled Water Splitting

Yuanzheng Long, Cheng Yang, Yulong Wu, Bohan Deng, Ziwei Li, Naveed Hussain, Kuangyu Wang, Ruyue Wang, Xian He, Peng Du, Zeliang Guo, Jialiang Lang, Kai Huang and Hui Wu**

Supporting Information

Cable-car electrocatalysis to drive fully decoupled water splitting

Yuanzheng Long, Cheng Yang, Yulong Wu, Bohan Deng, Ziwei Li, Naveed Hussain, Kuangyu Wang, Ruyue Wang, Xian He, Peng Du, Zeliang Guo, Jialiang Lang, Kai Huang and Hui Wu**

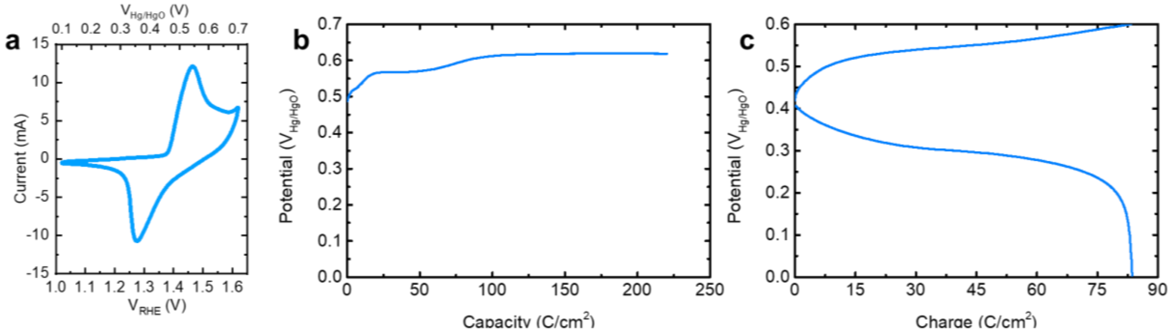

Figure S1. Electrochemistry performance of the $Ni(OH)_2$. (a) CV performance of $Ni(OH)_2$. (b) A long charge performance. (c) Charge and discharge performance.

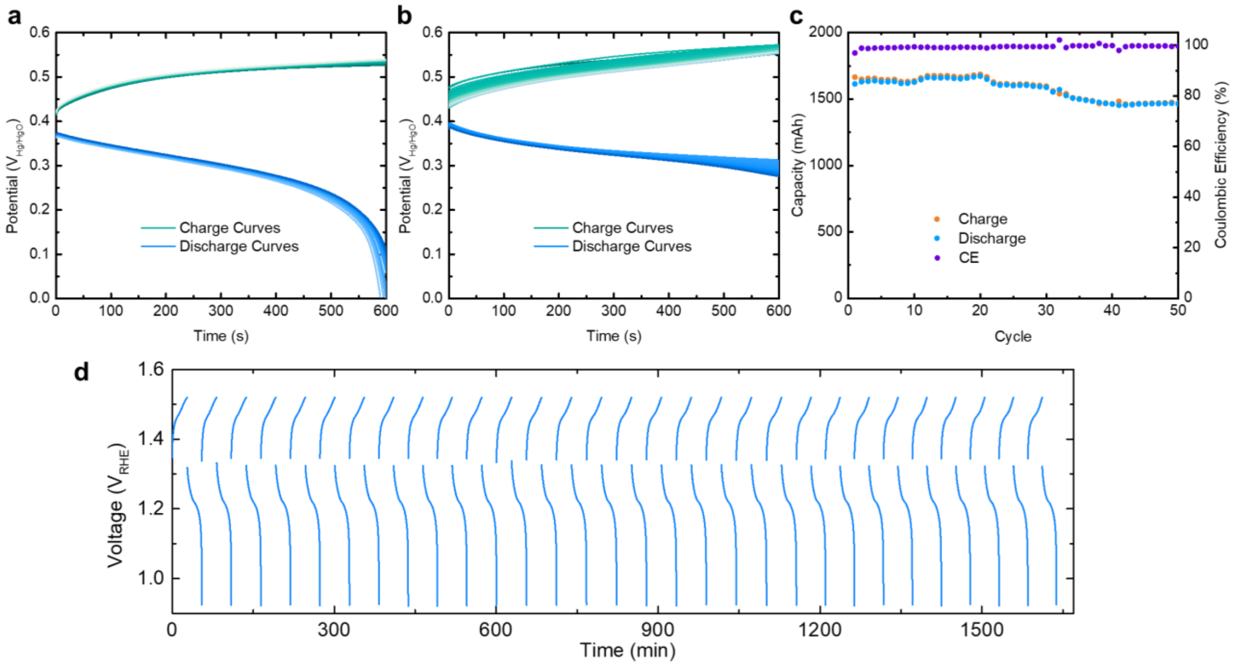

Figure S2. Cycling stability of charge/discharge cycles for a CCE. (a, d) Charge and fully discharge performance. (b) Charge and discharge performance are similar to the actual situation in the running CCE device. (c) Stability of the Coulombic Efficiency (CE) and capacity for 50 cycles.

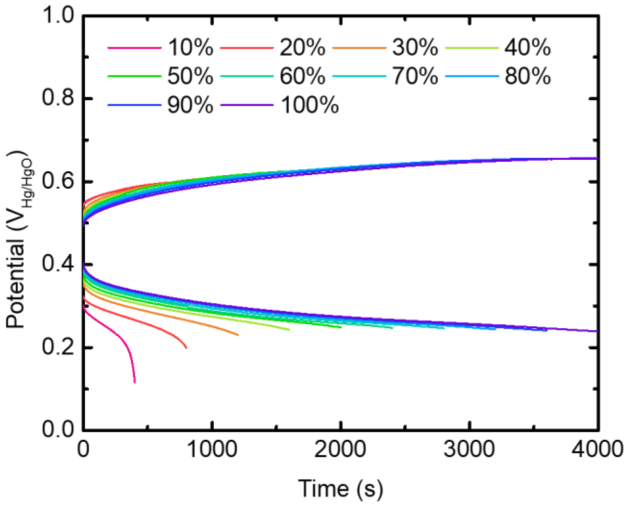

Figure S3. Charge and discharge performance of the CCE with different charged states. CCE with different states of charge can be discharged with a Coulombic efficiency of nearly 100 %.

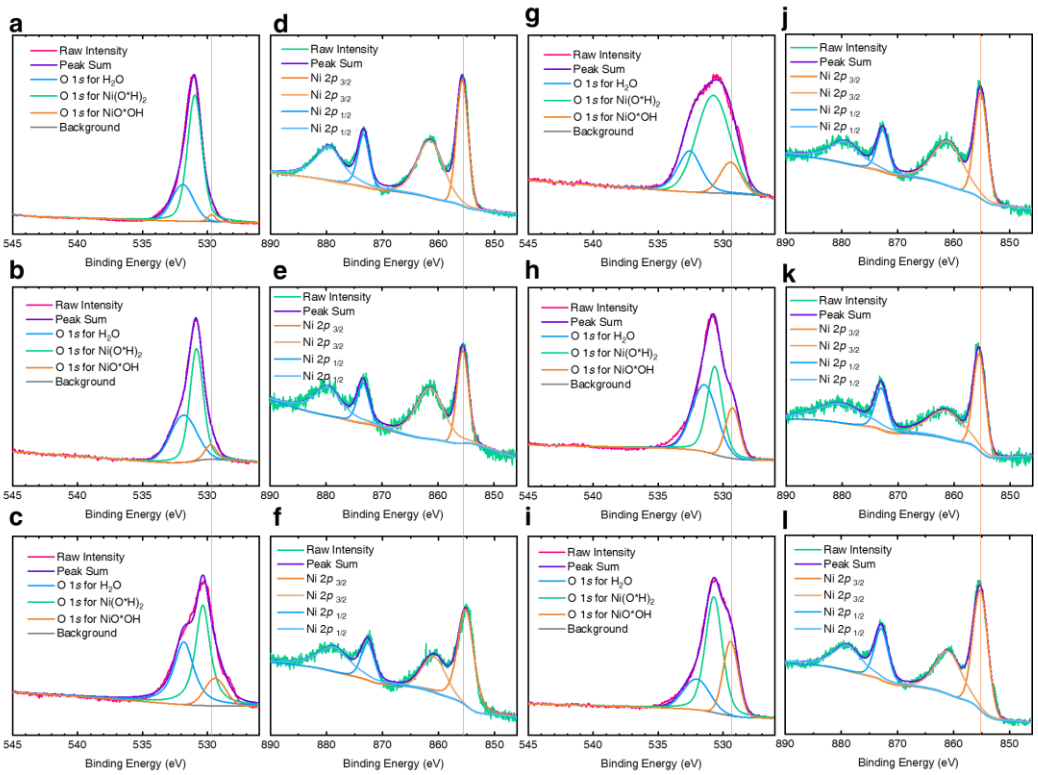

Figure S4. XPS of the CCE in different charged states.

O 1s (a)-(c), (g)-(i); Ni 2p (d)-(f), (j)-(l).

(a, d) pristine $Ni(OH)_2$ without charging.

(b, e) electrode charged ~25% of its capacity.

(c, f) electrode charged ~50% of its capacity.

(g, j) electrode charged ~75% of its capacity.

(h, k) electrode charged ~100% of its capacity.

(i, l) electrode overcharged to ~150% of its capacity.

The shifting and rising of the orange peaks prove the theory we apply.

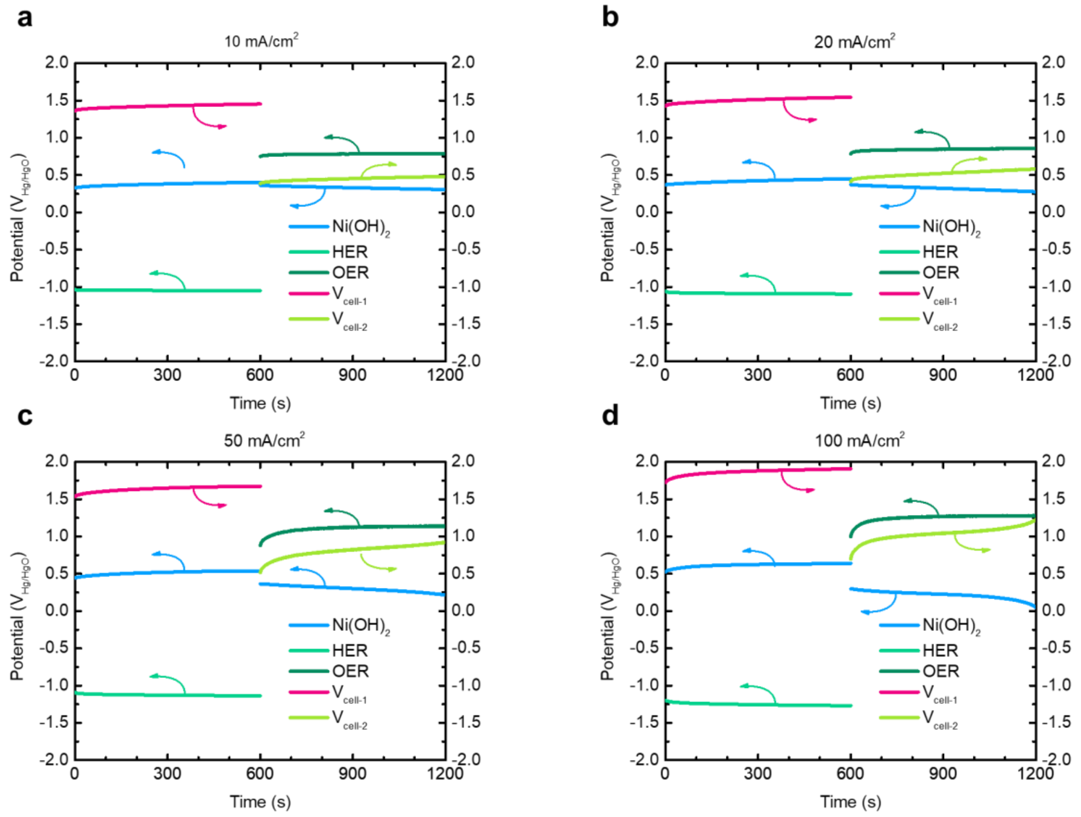

Figure S5. Potential relation of the carrier in different current densities. (a) 10 mA/cm^2; (b) 20 mA/cm^2 ; (c) 50 mA/cm^2 ; (d) 100 mA/cm^2

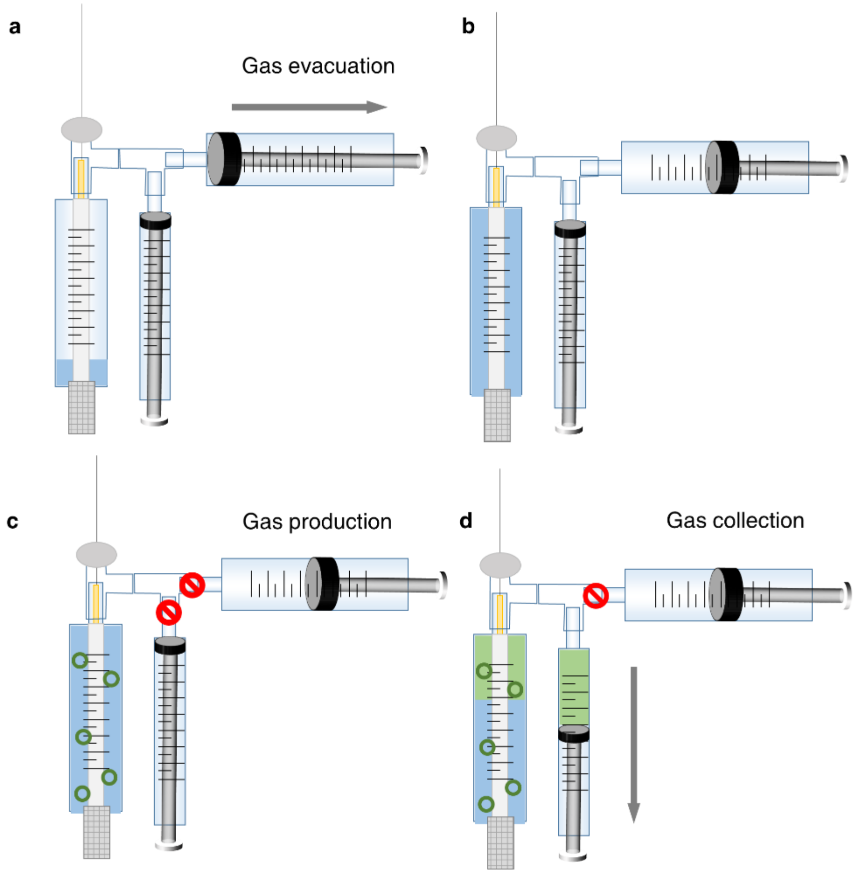

Figure S6. Schematic images of the hydrogen collection module. (a) (b) Gas evacuation to evacuate the remaining gas in the collecting syringe. (c) Hydrogen production from the HER electrode. (d) The gas sample is taken from the small syringe for further testing.

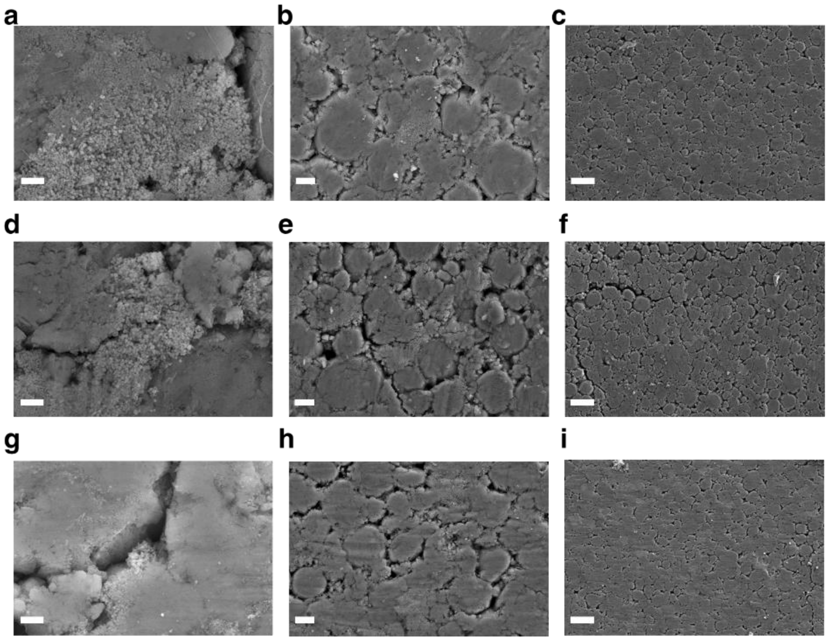

Figure S7. SEM images of the CCE in different states of charge. (a-c) CCE with 0% charged. (d-f) CCE with 50% charged. (g-i) CCE with 100% charged. The SEM images show that little difference happens in different states of charge. No obvious spalling is observed. Scale bar: (a, d, g) 1 μm; (b, e, h) 4 μm; (c, f, i) 20 μm.

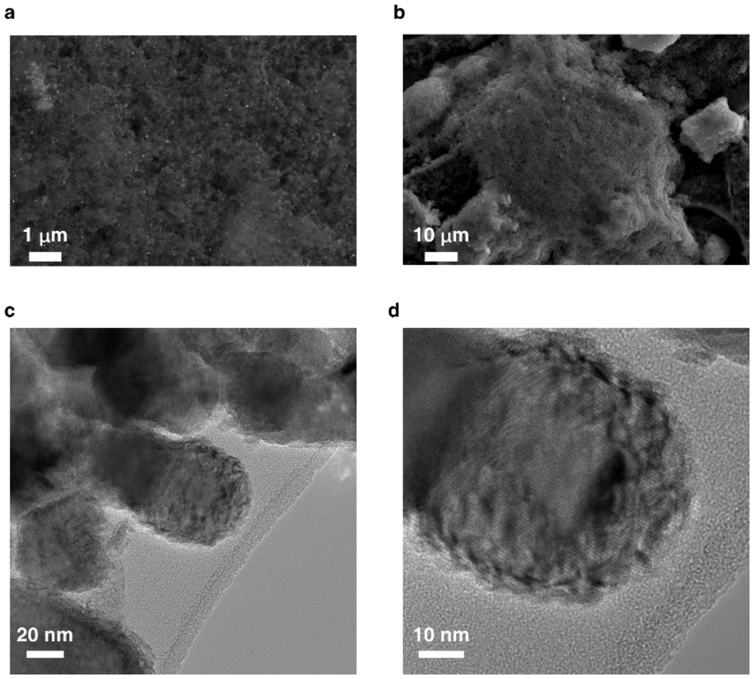

Figure S8. Morphology of electrodes. (a) SEM images of the OER electrode at low magnification. (b) SEM images of the OER electrode at high magnification. (c, d) TEM image of the $Ni(OH)_2$ particle on the CCE.

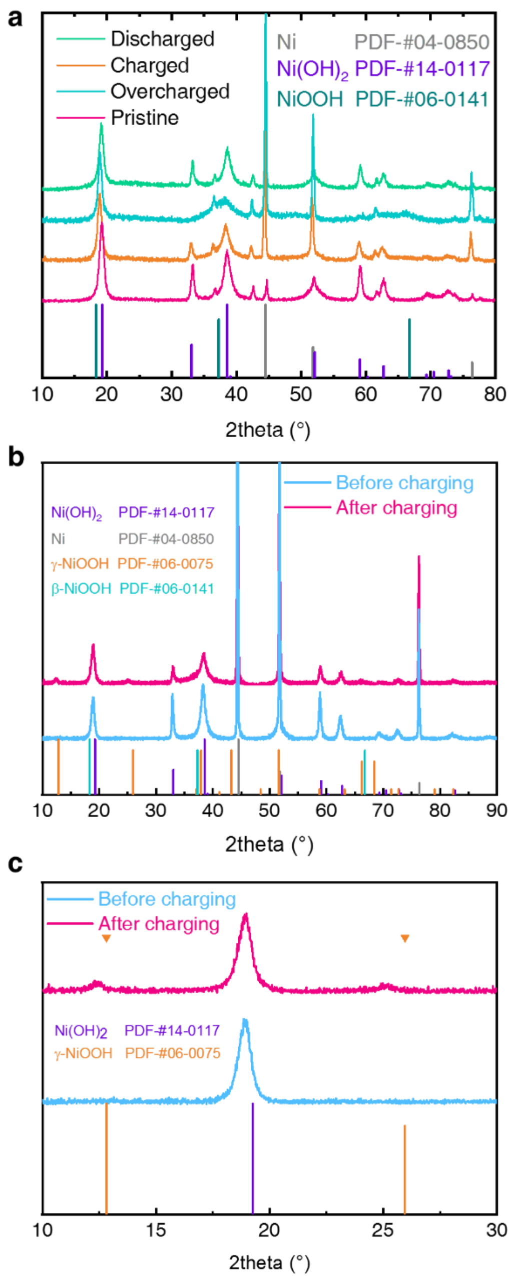

Figure S9. XRD patterns. (a) XRD patterns of CCEs at different charged states. XRD patterns show a left shift for charged and overcharged CCEs at the peak around 18°. The peak shifts right when the CCE is discharged. (b, c) XRD patterns of pristine and overcharged $Ni(OH)_2$ powder. b is the full pattern, and c is the detailed pattern where the peaks refer to the γ-NiOOH when overcharged.

Supplementary Videos

Supplementary Video 1. The operation of the CCE system continuously produces hydrogen. (played at 15x and 60x speed)

Supplementary Video 2. The close-up of the hydrogen generation module.
